# Molecular targeted photoimmunotherapy for HER2-positive human gastric cancer in combination with chemotherapy results in improved treatment outcomes through different cytotoxic mechanisms

**DOI:** 10.1186/s12885-016-2072-0

**Published:** 2016-01-25

**Authors:** Kimihiro Ito, Makoto Mitsunaga, Seiji Arihiro, Masayuki Saruta, Mika Matsuoka, Hisataka Kobayashi, Hisao Tajiri

**Affiliations:** Division of Gastroenterology and Hepatology, Department of Internal Medicine, The Jikei University School of Medicine, 3-25-8 Nishishinbashi, Minato, Tokyo 105-8461 Japan; Molecular Imaging Program, Center for Cancer Research, National Cancer Institute, NIH, Building 10, RoomB3B69, MSC1088, Bethesda, MD 20892-1088 USA

**Keywords:** Molecular targeted therapy, Photoimmunotherapy, HER2, Gastric cancer, 5-fluorouracil

## Abstract

**Background:**

Photoimmunotherapy (PIT) is a novel type of molecular optical imaging-guided cancer phototherapy based on a monoclonal antibody conjugated to a photosensitizer, IR700, in combination with near-infrared (NIR) light. PIT rapidly causes target-specific cell death by inducing cell membrane damages and appears to be highly effective; however, we have previously demonstrated that tumor recurrences were eventually seen in PIT-treated mice, likely owing to inhomogeneous mAb-IR700 conjugate distribution in the tumor, thus limiting the effectiveness of PIT as a monotherapy. Here, we examined the effects of human epidermal growth factor-2 (HER2)-targeted PIT in combination with 5-fluorouracil (5-FU) compared to PIT alone for HER2-expressing human gastric cancer cells.

**Methods:**

NCI-N87 cells, HER2-positive human gastric cancer cells, were used for the experiments. Trastuzumab, a monoclonal antibody directed against HER2, was conjugated to IR700. To assess the short-term cytotoxicity and examine the apoptotic effects upon addition of 5-FU in vitro, we performed LIVE/DEAD and caspase-3 activity assays. Additionally, to explore the effects on long-term growth inhibition, trypan blue dye exclusion assay was performed. NCI-N87 tumor xenograft models were prepared for in vivo treatment studies and the tumor-bearing mice were randomized into various treatment groups.

**Results:**

Compared to PIT alone, the combination of HER2-targeted PIT and 5-FU rapidly induced significant cytotoxicity in both the short-term and long-term cytotoxicity assays. While both 5-FU and/or trastuzumab-IR700 conjugate treatment induced an increase in caspase-3 activity, there was no additional increase in caspase-3 activity upon NIR light irradiation after incubation with 5-FU and/or trastuzumab-IR700. The combination of HER2-targeted PIT and 5-FU resulted in greater and longer tumor growth inhibition than PIT monotherapy in vivo. This combined effect of PIT and 5-FU is likely owing to their different mechanisms of inducing tumor cell death, namely necrotic membrane damage by PIT and apoptotic cell death by 5-FU and trastuzumab.

**Conclusions:**

PIT in combination with 5-FU resulted in enhanced antitumor effects compared to PIT alone for HER2-expressing human gastric cancer in vitro and in vivo. This combination photoimmunochemotherapy represents a practical method for treating human gastric cancer and should be investigated further in the clinical setting.

**Electronic supplementary material:**

The online version of this article (doi:10.1186/s12885-016-2072-0) contains supplementary material, which is available to authorized users.

## Background

Gastric cancer is the fourth most commonly diagnosed cancer and the second leading cause of cancer-related death worldwide. Moreover, gastric cancer is associated with a poor prognosis [1–3]. Conventional cancer therapies, such as surgery, radiation, and chemotherapy, can cause damage or toxicity to both the adjacent and distant normal tissues, which in turn limits the effectiveness of these therapies [4, 5]. To achieve more effective cancer control, as well as to minimize the side effects associated with conventional cancer therapies, molecular targeted cancer therapies, such as monoclonal antibodies and tyrosine kinase inhibitors, have been developed in recent years [4, 6].

Overexpression of human epidermal growth factor receptor 2 (HER2), a member of the epidermal growth factor receptor family, which is known to play roles in cell proliferation, differentiation, and apoptosis [7, 8], has been previously reported in gastric cancer, as well as in various other cancers [9, 10]. Accordingly, trastuzumab, a humanized mAb directed against HER2, has been demonstrated to induce antitumor effects in HER2-positive gastric cancer, both alone and in combination with various chemotherapeutic agents [9, 11].

Photoimmunotherapy (PIT) is a newly described cancer theranostic system based on a molecular-targeted mAb conjugated to a photosensitizer, IRDye700DX (IR700), together with irradiation of near-infrared (NIR) light (peak at 689 nm) [12]. When exposed to NIR light, the mAb-IR700 conjugate is activated; however, it only shows selective activity against cells to which the conjugate is specifically bound and fluorescent, whereas it has no effect on the cells that do not express that particular molecular target [12–14]. NIR light exposure with mAb-IR700 conjugate leads to rapid and target-selective cell death, mainly as a result of necrosis, in vitro [12], and tumor shrinkage can be obtained even with a single administration of mAb-IR700 followed by a single exposure to NIR light in vivo [12–14]. However, although PIT has been demonstrated to be highly effective, our previous study showed that a subset of cancer cells survived after PIT in an in vivo mouse model, and tumor recurrences were eventually seen in the treated mice as a result of inhomogeneous mAb-IR700 distribution in the targeted tumor [15]. Moreover, tumor heterogeneity is relatively common in human epithelial carcinomas, including in gastric cancer [9, 16, 17], and this may limit the effects of PIT treatment.

Here, we investigated whether the antitumor effect of combined HER2-targeted PIT with conventional chemotherapy was enhanced compared to treatment with PIT alone in HER2-expressing human gastric cancer in vitro and in vivo.

## Methods

### Reagents

Trastuzumab (Herceptin®), a mAb directs against the extracellular domain of HER2, was purchased from Chugai Pharmaceutical Co., Ltd. (Tokyo, Japan). IRDye700DX N-hydroxysuccinimide ester (IR700; a photosensitizer) was purchased from LI-COR Biosciences (Lincoln, NE). The chemotherapeutic agent 5-fluorouracil (5-FU), which is widely used as a standard treatment for human gastric cancer [18], was purchased from Kyowa Hakko Kirin Co., Ltd. (Tokyo, Japan).

### Synthesis and purification of IR700-conjugated trastuzumab

Trastuzumab (1.0 mg, 6.8 nmol) was incubated with IR700 (66.8 μg, 34.2 nmol) in 0.1 M Na_2_HPO_4_ (pH 8.5) at room temperature for 1 h. The mixture was purified using a Sephadex G50 column (PD-10; GE Healthcare, Wauwatosa, WI). The concentrations of IR700 and protein were measured by absorption reading using spectroscopy (UV-1800; Shimadzu corp., Kyoto, Japan) to confirm the number of fluorophore molecules conjugated to each trastuzumab molecule. The number of IR700 molecules per trastuzumab molecule was adjusted to approximately 3.

### Cell lines and culture conditions

Two human gastric cancer cell lines were used in this study. HER2-expressing NCI-N87 cells were purchased from American Type Culture Collection (Manassas, VA), and HER2-negative MKN-45 cells were purchased from RIKEN Cell Bank (Tsukuba, Japan). Both cell lines were cultured in Roswell Park Memorial Institute (RPMI) 1640 medium supplemented with 10 fetal bovine serum and 1 % penicillin-streptomycin (Life technologies, Gaithersburg, MD) in a humidified incubator at 37 °C in an atmosphere of 95 air and 5 % carbon dioxide (CO_2_). The study was carried out in accordance with the Helsinki declaration.

### Cytotoxicity assay for human gastric cancer cells by 5-fluorouracil

To determine the 50 % inhibitory concentration (IC_50_) of 5-FU for NCI-N87 and MKN-45 cells, we performed 3-(4,5-dimethylthiazol-2-yl)-5-(3-carboxymethoxyphenyl)-2-(4-sulfophenyl)-2H-tetrazolium, inner salt (MTS) assay. The cells were seeded at 1 × 10^4^ cells per well in a 96-well microplate, and exposed to various concentrations of 5-FU (0, 0.03, 0.1, 0.3, 1, 3, 10, 30, 100, 300 and 1000 μΜ) for 48 h at 37 °C in 5 % CO_2_. After the treatments, CellTiter 96® Aqueous One Solution (Promega, Madison, WI) was added to each well, followed by incubation for 2 h at 37 °C in 5 % CO_2_, and the absorbance of each well was measured using a microplate reader (iMark, Bio-Rad, Hercules, CA) at 540 nm.

### Determination of HER2 expression for human gastric cancer cells in vitro

To examine the HER2 expression in NCI-N87 and MKN-45 cells, IR700 fluorescence was analyzed by flow cytometry (MACSQant analyzer; Miltenyi Biotec, Bergisch Gladbach, Germany), and the mean fluorescence intensities were evaluated. The cells were seeded in 35-mm dishes at 5 × 10^5^ cells/dish and cultured for 48 h at 37 °C in 5 % CO_2_. After 3 h incubation with 10 μg/ml of trastuzumab-IR700 conjugate (Tra-IR700), the media were removed and the plates were washed with phosphate-buffered saline (PBS); subsequently, flow cytometric analysis was performed. In addition, to confirm the target specificity, unconjugated trastuzumab (100 μg/ml) was added to the cells to block the HER2 molecules before incubation with the Tra-IR700 conjugate. The mean fluorescence intensities were calculated and compared to the isotype control. Furthermore, fluorescence microscopy was performed using an IX73 fluorescence microscope (Olympus, Tokyo, Japan) to determine the HER2-specific binding of Tra-IR700 and the subcellular localization of IR700.

### Cytotoxicity assay in response to NIR light irradiation and 5-fluorouracil in vitro

To assess the early stage of cytotoxicity in response to PIT and 5-FU treatment, we used the LIVE/DEAD® Fixable Green Dead Cell Stain Kit (Life Technologies), which can detect damaged cellular membranes by flow cytometric analysis. NCI-N87 and MKN-45 cells were seeded in 35-mm dishes at 5 × 10^5^ cells/dish and cultured for 48 h at 37 °C in 5 % CO_2_. The following treatment groups were analyzed: (1) PBS without NIR light irradiation (negative control), (2) IC_50_ of 5-FU without NIR light irradiation, (3) 10 μg/ml of Tra-IR700 without NIR light irradiation, (4) IC_50_ of 5-FU and 10 μg/ml of Tra-IR700 without NIR light irradiation, (5) 10 μg/ml of Tra-IR700, followed by NIR light irradiation, and (6) IC_50_ of 5-FU and 10 μg/ml of Tra-IR700, followed by NIR light irradiation. After 24 h incubation with 5-FU and/or Tra-IR700, the medium was removed and replaced by phenol red-free RPMI 1640 after washing with PBS. Subsequently, NIR light (1 J/cm^2^, 2 J/cm^2^, or 4 J/cm^2^) was irradiated to the cells. After the treatments, the cells were collected in PBS, and LIVE/DEAD green fluorescent reactive dye was added to the cell suspension. After 30 min incubation at room temperature, the cells were analyzed by flow cytometry.

To further explore the long-term growth inhibition, we performed trypan blue dye exclusion assay. The cells were seeded at 2 × 10^5^ cells in 35-mm dishes and cultured for 24 h at 37 °C in 5 % CO_2._ The IC_50_ of 5-FU and/or 10 μg/ml of Tra-IR700 were added to the medium and incubated for another 24 h. Subsequently, the medium was removed and replaced by phenol red-free RPMI 1640 after washing with PBS, and NIR light was irradiated to the cells. At the indicated time point after NIR light irradiation, the cells were collected, and the viable cells were counted based on the trypan blue dye uptake.

### Caspase-3 activity assay

To examine the apoptotic effects in response to each individual treatment or combination thereof, we measured the activity of caspase-3 using the Caspase-3 colorimetric assay kit (MBL, Nagoya, Japan). The cells were treated using the same protocol as for the LIVE/DEAD assay. After the treatments, the cells were washed with PBS and lysed in cell lysis buffer for 10 min on ice, after which the cell lysates were centrifuged at 10,000 *g* for 5 min and the protein concentrations of the supernatants were estimated using the bicinchoninic acid (BCA) protein assay kit (Thermo Fisher Scientific, Waltham, MA). Equal amounts of protein extracts were incubated overnight with a reaction buffer containing dithiothreitol and caspase-3 substrates in a 96-well microplate at 37 °C. The absorbance at 405 nm was measured using a microplate reader.

### HER2-specific accumulation of Tra-IR700 in vivo

Female 6-week-old BALB/c-nu/nu mice (CAnN.Cg-Foxn1 < nu>/CrlCrlj nu/nu) were obtained from Charles River Laboratories Japan, Inc. (Yokohama, Japan). All mice were allowed to acclimatize and recover from shipping-related stress for 1 week before the study, and were kept in a controlled light–dark cycle (12 h-12 h) environment. All animal experiments were conducted in accordance with the guidelines established by the Animal Care Committee of the Jikei University School of Medicine. To examine Tra-IR700 distribution in vivo, we prepared tumor xenograft models bearing NCI-N87 and MKN-45 tumors. A total of 5 × 10^6^ NCI-N87 cells were injected subcutaneously into the right dorsum of each mouse, and 3 × 10^6^ MKN-45 cells were injected subcutaneously into the left dorsum of the same mouse. The tumor xenografts were measured with a caliper, and the tumor volume was calculated using the following formula: length × width × height × 0.5 [19]. When each tumor reached approximately 15 mm^3^, 50 μg of Tra-IR700 was injected intravenously. Distribution of IR700 fluorescence was evaluated using the IVIS^®^ Imaging System (Caliper Life Sciences, Hopkinton, MA). Fluorescence images were obtained 1, 2, 3, and 5 days after Tra-IR700 injection under the same settings (e.g. exposure time, camera binning, and stage height) using isoflurane anesthesia. All fluorescence images were analyzed with Image J software (http://rsb.info.nih.gov/ij/; National Institutes of Health, Rockville, MD). The region of interest was manually determined on each tumor area depending on where the IR700 fluorescence was localized. The background regions, each being approximately the same region size as that of the tumor regions, were subtracted from the tumor regions in the same mouse.

### HER2 targeting photoimmunochemotherapy with Tra-IR700 and 5-FU in vivo

To determine the antitumor effects of PIT in combination with 5-FU compared to PIT alone in vivo, the following experiments were conducted. A total of 5 × 10^6^ NCI-N87 cells were injected subcutaneously into the right dorsum of the mice, and the tumor xenografts were measured with a caliper 3 times per a week, as described above. Tumors reaching approximately 15 mm^3^ in volume were selected and randomized into the following 8 groups (at least *n* = 10 mice in each group): (a) no treatment, (b) 30 μg/g/day of 5-FU i.p. without NIR light irradiation, (c) 300 μg of Tra-IR700 i.v. without NIR light irradiation, (d) NIR light irradiation (100 J/cm^2^) without 5-FU or Tra-IR700 injection, (e) 30 μg/g/day of 5-FU i.p. and 300 μg of Tra-IR700 i.v. without NIR light irradiation, (f) 30 μg/g/day of 5-FU i.p. followed by NIR light irradiation (100 J/cm^2^), (g) 300 μg of Tra-IR700 i.v. followed by NIR light irradiation (100 J/cm^2^), and (h) 30 μg/g/day of 5-FU i.p. and 300 μg of Tra-IR700 i.v. followed by NIR light irradiation (100 J/cm^2^). Tra-IR700 injection was performed at the time of mouse randomization (Day 0), and NIR light irradiation was performed for Tra-IR700 localizing tumors detected by the in vivo imaging system 24 h after injection (Day 1). Subsequently, 5-FU was injected on 3 consecutive days (Day 1 [just after NIR light irradiation], Day 2 and Day 3; total amount: 90 μg/g) (Fig. 4a). All treatments were performed using isoflurane anesthesia. After the treatments, the tumor xenografts continued to be measured 3 times a week and the mice were monitored for 30 days.

### Histological analysis

To evaluate the histological changes after the treatments, the mice were euthanized at the indicated time points and the NCI-N87 tumors were harvested and fixed in 10 % formalin for 24 h. Serial 10-μm slice sections were stained with hematoxylin and eosin.

### Statistical analysis

Data are expressed as means ± SEM from a minimum of three experiments. Statistical analyses were carried out using GraphPad Prism software (GraphPad Software Inc., La Jolla, CA). For in vitro experiments, Student’s *t* test was used to compare the two treatment groups. For in vivo experiments, the Mann–Whitney U test was used to evaluate the differences in tumor volume. *P* < 0.05 was considered to indicate a statistically significant difference.

## Results

### HER2 expression of human gastric cancer cells

**Fig. 1:**
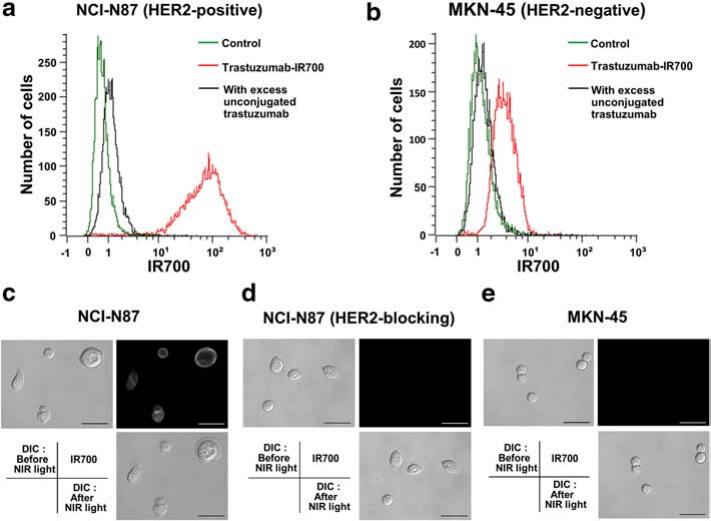
In vitro human epidermal growth factor receptor 2 (HER2) expression in human gastric cancer cells. **a**, **b** Flow cytometry revealed strong HER2-specific signals in NCI-N87 cells and very weak signals in MKN-45 cells. Specific binding of trastuzumab-IR700 was demonstrated by excess trastuzumab blocking. **c**, **d**, **e** Fluorescence microscopy showed HER2-specific IR700 localization in NCI-N87 cells. Conversely, IR700 fluorescence was not detected in MKN-45 cells or in NCI-N87 cells pre-treated with an excess volume of trastuzumab. Cellular swelling and bleb formation were rapidly induced after excitation light in NCI-N87 cells but not in MKN-45 cells (lower right). Scale bar, 50 μm. DIC: differential interference contrast, NIR: near-infrared

In vitro HER2 expression was determined by flow cytometric analysis and fluorescence microscopy. In NCI-N87 and MKN-45 cells, the ratios of the mean fluorescence intensity (MFI) compared to the isotype control were 154.7 ± 15.4 and 3.7 ± 0.4, respectively (*n* = 3 each) (Fig. 1a, b). When pre-incubated with 100 μg of unconjugated trastuzumab to block the HER2 molecules, the signal from Tra-IR700 was completely blocked in both the NCI-N87 and MKN-45 cells (Fig. 1a, b); the ratios of the MFI compared to the isotype control in the NCI-N87 and MKN-45 cells were 7.4 ± 2.7 and 1.9 ± 0.3, respectively (*n* = 3 each). As only very weak HER2 expression was found in the MKN-45 cells, we considered MKN-45 cells as a HER2-negative control in this study. Fluorescence images were obtained to confirm HER2-specific IR700 localization during the irradiation of NIR light. IR700 fluorescence was detected on the surface and cytoplasmic vesicles in HER2-expressing NCI-N87 cells after 3 h incubation with Tra-IR700, while MKN-45 cells did not show detectable IR700 fluorescence under the same image acquisition conditions (Fig. 1c, e). While excitation light irradiation (692 ± 20 nm, power density; 24 mW/cm^2^) to NCI-N87 cells rapidly induced cellular swelling and surface bleb formation (Fig. 1c), immediate cell death was not induced when the cells were pre-incubated with excess unconjugated trastuzumab (Fig. 1d). On the other hand, the HER2-negative MKN-45 cells did not show any morphological changes upon NIR light irradiation (Fig. 1e).

### In vitro photoimmunochemotherapy for human gastric cancer cells

**Fig. 2:**
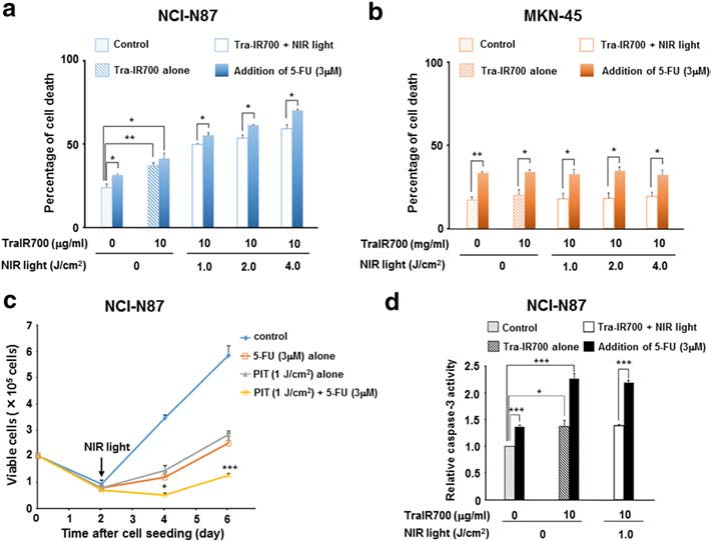
In vitro photoimmunochemotherapy for human gastric cancer cells. **a**, **b** Human epidermal growth factor receptor 2 (HER2)-targeting photoimmunotherapy (PIT) induced near-infrared (NIR) light dose-dependent cell death in NCI-N87 cells but not in MKN-45 cells. The percentage of cell death was significantly increased by PIT treatment in combination with 5-fluorouracil (5-FU) compared to PIT alone. Significant cytotoxic effects of 5-FU were observed in both NCI-N87 and MKN-45 cells. Spotted bar: no treatment, oblique line bar: trastuzumab-IR700 (Tra-IR700) (10 μg/ml) alone, unfilled bar: Tra-IR700 (10 μg/ml) followed by NIR light irradiation (PIT alone), filled bar: addition of 5-FU (3 μM). Data are presented as means ± SEM (*n* = 3, *P < 0.05, **P < 0.01, Student’s *t* test). **c** Significant long-term growth inhibition was observed in cells treated by PIT and 5-FU, as determined by the trypan blue exclusion assay. Data are presented as means ± SEM (*n* = 3, *P < 0.05, ***P < 0.001 vs. treated with PIT alone, Student’s *t* test). D While 5-FU and/or Tra-IR700 treatment induced an increase in caspase-3 activity, NIR light irradiation (PIT treatment) did not further increase the caspase-3 activity in NCI-N87 cells. Data are presented as means ± SEM (*n* = 3, *P < 0.05, ***P < 0.001, Student’s *t* test). Spotted bar: no treatment, oblique line bar: Tra-IR700 (10 μg/ml) alone, unfilled bar: Tra-IR700 (10 μg/ml) followed by NIR light irradiation (PIT alone), filled bar: addition of 5-FU (3 μM)

We initially determined the cytotoxic effect of 5-FU for each gastric cancer cell line using the MTS assay. The IC_50_ of 5-FU was found to be 3.3 ± 1.5 μM in NCI-N87 cells and 3.1 ± 0.5 μΜ in MKN-45 cells (*n* = 3 each). We used the IC_50_ of 5-FU as the therapeutic dose for the cells. Previous studies have shown that tumor cell death in response to PIT is rapidly induced through membranous damage, as determined by the LIVE/DEAD assay [12–14]. Accordingly, we here employed the same assay to assess the cytotoxic effects of combination treatment with PIT and 5-FU. We observed significant differences in the percentages of cell death between 5-FU alone and untreated controls in both NCI-N87 (*P* = 0.0336) (Fig. 2a) and MKN-45 cells (*P* = 0.0013) (Fig. 2b). Moreover, we observed significant cytotoxicity associated with exposure to Tra-IR700 without NIR light compared to the untreated controls in NCI-N87 cells (*P* = 0.0088), but not in MKN-45 cells. In NCI-N87 cells, the percentage of cell death in response to PIT was significantly increased compared to the untreated control in an irradiation dose-dependent manner (*P* < 0.001 for all irradiated doses), while no cytotoxicity associated with PIT was seen in MKN-45 cells. In addition, the percentage of cell death in the cells treated with PIT in combination with 5-FU compared to the cells treated with PIT alone was significantly increased at all of the irradiated NIR light doses in NCI-N87 cells (PIT 1 J/cm^2^ + 5-FU vs. PIT 1 J/cm^2^: *P* = 0.0478; PIT 2 J/cm^2^ + 5-FU vs. PIT 2 J/cm^2^: *P* = 0.0175; and PIT 4 J/cm^2^ + 5-FU vs. PIT 4 J/cm^2^: *P* = 0.0113; Fig. 2a), while no combined effect was observed in MKN-45 cells (Fig. 2b). We also determined the long-term treatment effects of PIT and 5-FU using the trypan blue dye exclusion assay. As shown in Fig. 2c, significant growth inhibition was observed in the cells treated with PIT in combination with 5-FU compared to PIT alone (4 days after NIR light irradiation: *P* < 0.001). To determine the mechanism of the cytotoxicity induced by PIT and/or 5-FU treatment, we next examined the apoptotic effects of these treatments using the caspase-3 activity assay in NCI-N87 cells. While a significant increase in caspase-3 activity was found when the cells were treated with 5-FU or Tra-IR700 (without NIR light), NIR light irradiation did not further increase the caspase-3 activity (Fig. 2d).

### HER2-specific accumulation of trastuzumab-IR700 conjugate in vivo

**Fig. 3:**
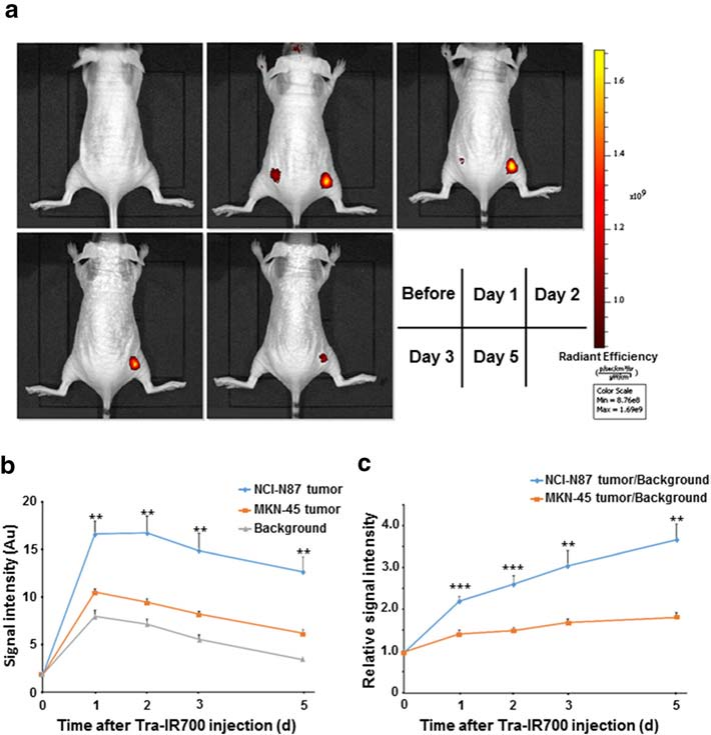
Human epidermal growth factor receptor 2 (HER2)-specific accumulation of trastuzumab-IR700 conjugate (Tra-IR700) in vivo. **a** Tra-IR700 distribution over time was assessed using an in vivo imaging system. NCI-N87 tumors (right dorsum) were clearly and selectively visualized by IR700 fluorescence as early as 1 day after Tra-IR700 injection. Conversely, MKN-45 tumors (left dorsum) were only weakly visualized by IR700 fluorescence, and the signals gradually attenuated over time (*n* = 5 mice). **b** Fluorescence intensity of IR700 in NCI-N87 tumors, MKN-45 tumors, and background. Data are presented as means ± SEM (*n* = 5 mice, ** *P* < 0.01 vs. MKN-45 tumors, Student’s *t* test). **c** Tumor-to-background ratios of IR700 fluorescence intensity in NCI-N87 and MKN-45 tumors. Data are presented as means ± SEM (*n* = 5 mice, ** *P* < 0.01, *** *P* < 0.001 vs. MKN-45 tumors, Student’s *t* test)

We next examined the biodistribution of Tra-IR700 in the tumor xenograft models by using an in vivo fluorescence imaging system. The NCI-N87 tumors were specifically visualized with IR700 fluorescence on Day 1 after intravenous injection of Tra-IR700. The highest signal was observed between Days 1 and 2 after Tra-IR700 injection, and the signal gradually decreased thereafter (Fig. 3a, b). As shown in Fig. 3c, the tumor-to-background ratios increased over time in NCI-N87 tumors. In contrast, upon visualization of the MKN-45 tumors using IR700 fluorescence on Day 1 after injection of Tra-IR700, the signals were significantly weaker than those of the NCI-N87 tumors (*n* = 5 mice, *P* = 0.0024; Fig. 3b), and the IR700 signal subsequently decreased. Except for the HER2-positive tumors, no other IR700 localization was found.

### In vivo photoimmunochemotherapy for human gastric cancer xenografts

**Fig. 4:**
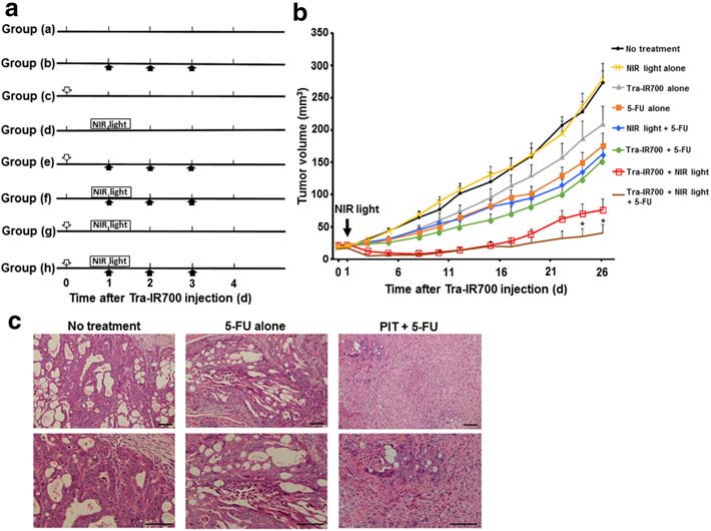
In vivo photoimmunochemotherapy for human gastric cancer xenografts. **a** Experimental protocol of photoimmunochemotherapy in vivo. (a) No treatment, (b) 30 μg/g/day of 5-fluorouracil (5-FU) i.p. without near-infrared (NIR) light irradiation, (c) 300 μg of trastuzumab-IR700 conjugate (Tra-IR700) i.v. without NIR light irradiation, (d) NIR light irradiation (100 J/cm^2^) without 5-FU or Tra-IR700 injection, (e) 30 μg/g/day of 5-FU i.p. and 300 μg of Tra-IR700 i.v. without NIR light irradiation, (f) 30 μg/g/day of 5-FU i.p. followed by NIR light irradiation (100 J/cm^2^), (g) 300 μg of Tra-IR700 i.v. followed by NIR light irradiation (100 J/cm^2^), and (h) 30 μg/g/day of 5-FU i.p. and 300 μg of Tra-IR700 i.v. followed by NIR light irradiation (100 J/cm^2^). Tra-IR700 injection was performed at the time of mouse randomization (Day 0), and NIR light irradiation was performed 24 h after Tra-IR700 injection (Day 1). Injection of 5-FU was performed on 3 consecutive days (Day 1 [just after NIR light irradiation], Day 2, and Day 3; total amount, 90 μg/g). Open downwards arrow: 300 μg of Tra-IR700 i.v.; filled upwards arrow: 30 μg/g of 5-FU i.p. **b** Photoimmunochemotherapy resulted in marked antitumor effects compared to photoimmunotherapy (PIT) alone. A significant therapeutic effect was observed in mice treated with PIT in combination with 5-FU compared to in mice treated with PIT alone on Days 24 and 26 after the initial treatment. Data are presented as means ± SEM (at least *n* = 10 mice in each group, * *P* < 0.05 vs. treated with PIT alone, Mann–Whitney U test). **c** Histological observations of the negative control and treated NCI-N87 tumors (*n* = 3 mice, hematoxylin and eosin staining). Bleeding and massive granulation with inflammatory changes were observed, and only scant viable cells were present in the tumor nodules treated with PIT in combination with 5-FU. Scale bar, 100 μm

The tumor xenografts reached 15 mm^3^ in volume approximately six days after subcutaneous injection of the NCI-N87 cells, after which time the mice were subsequently randomized into 8 groups. The NCI-N87 tumors were irradiated with a single dose of NIR light on Day 1 after Tra-IR700 injection, as the highest IR700 accumulation was observed at that time point. As our previous studies and preliminary results indicated that one-time injection of 300 μg antibody-IR700 conjugate followed by 100 J/cm^2^ NIR light irradiation was able to be achieved safely without any significant side effects [12–14], this condition was employed as the PIT treatment to assess the combination treatment effects of PIT and 5-FU. As compared with the various controls, apparent tumor growth inhibition was observed upon treatment with Tra-IR700 injection followed by NIR light irradiation after a few days. Importantly, tumor growth was significantly suppressed in the PIT and 5-FU treatment group compared to the PIT alone group (24 and 26 days after Tra-IR700 injection: *P* = 0.0415 and *P* = 0.0414, respectively) (Fig. 4b). Pathological analysis revealed cellular degeneration with nuclear swelling in the tumor nodules treated with 5-FU alone; on the other hand, massive granulation with inflammatory changes and scant viable cells were observed in the tumor nodules treated with PIT in combination with 5-FU (Fig. 4c).

## Discussion

PIT is a highly selective cancer therapy, which is based on a molecular-targeted mAb conjugated to the photosensitizer IR700 and the use of NIR light. We have previously reported that HER2 target-specific cell death was achieved with a single dose of Tra-IR700 administration followed by NIR light irradiation. However, some cancer cells survived after PIT, and recurrences eventually developed in our in vivo model [12, 13]. Generally, mAb distribution is heterogeneous in most tumors, especially in peripheral locations where the tumors receive good blood supply [13, 20–22]. In addition, tumor heterogeneity is relatively common in human epithelial carcinomas, including in gastric cancer [9, 16, 17]. Taken together, this suggests that the antitumor effects of PIT alone on human gastric cancer may be limited. To solve this problem, we here examined whether the antitumor effect was enhanced by combining PIT with chemotherapy, as compared with PIT alone, in human gastric cancer cells. Our findings suggest that PIT in combination with 5-FU showed enhanced antitumor effects in HER2-expressing human gastric cancer through different cytotoxic mechanisms, both in vitro and in vivo.

Flow cytometric analyses revealed that the relative MFI from the Tra-IR700 signal compared to the isotype control was much higher in NCI-N87 cells, whereas MKN-45 cells showed a very low relative MFI (Fig. 1a, b). In both cell lines, complete signal blocking was induced by adding excess unconjugated trastuzumab, indicating that the cellular membrane binding of Tra-IR700 was HER2-specific. Moreover, fluorescence microscopy clearly detected IR700 fluorescence after incubation with Tra-IR700 for 3 h in NCI-N87 cells, but not in MKN-45 cells. Thus, as the HER2 expression in MKN-45 cells was determined to be very weak, we considered MKN-45 cells as a HER2-negative control in this study. Consistent with previous reports, necrotic cell death was rapidly induced in NCI-N87 cells treated with Tra-IR700 upon NIR light irradiation, as demonstrated by bleb formation and cellular swelling. On the other hand, MKN-45 cells did not show any morphological changes (Fig. 1c, e), indicating that target-specific membrane binding of Tra-IR700 was required to induce phototoxicity.

Significant cytotoxicity due to treatment with the IC_50_ of 5-FU was observed in the LIVE/DEAD assay in both NCI-N87 and MKN-45 cells, supporting the hypothesis that 5-FU induces cell membrane damage by inhibiting DNA synthesis during apoptosis [23, 24] (Fig. 2a, b). In contrast, we observed significant cytotoxicity treated with Tra-IR700 compared to the untreated controls only in NCI-N87 cells, and there was no significant differences in the percentage of cell death between treated with Tra-IR700 and treated with unconjugated trastuzumab (Additional file 1: Figure S1). These results suggested that Tra-IR700 retained the same immunoreactivity as native unconjugated trastuzumab for NCI-N87 cells. There was a significant increase in caspase-3 activity when NCI-N87 cells were treated with 5-FU and/or Tra-IR700 without NIR light, however, NIR light irradiation after incubation with Tra-IR700 or Tra-IR700 in combination with 5-FU did not result in any additional increases in caspase-3 activity not only just after NIR light irradiation but also 24 h after the irradiation (Additional file 1: Figure S2). These results indicate that 5-FU and/or Tra-IR700 treatment induces apoptosis, while PIT treatment (NIR light with Tra-IR700) does not induce additional apoptosis. Taken together with these results, rapid necrotic cell death in response to NIR light (with Tra-IR700) in addition to apoptotic cell death in response to 5-FU and Tra-IR700 treatment play major roles in this combination treatment.

Additionally, long-term cytotoxicity was assessed by the trypan blue exclusion assay, which confirmed the enhanced treatment effect of PIT and 5-FU combination treatment in NCI-N87 cells (Fig. 2c).

The in vivo biodistribution study by detecting IR700 fluorescence revealed that HER2-positive NCI-N87 tumors specifically showed IR700 fluorescence starting at Day 1 after intravenous injection of Tra-IR700, and this signal continued to increase until Day 2 after injection. In these tumors, the tumor-to-background ratio was increased over time, likely owing to differences in the clearance between the antibody-specific binding and nonspecific blood pool. In contrast, IR700 fluorescence in HER2-negative MKN-45 tumors, as well as in the background normal tissues, was observed for up to 1 day after injection, and thereafter gradually decreased. These findings suggest that HER2-specific IR700 fluorescence persists through specific membrane binding with trastuzumab, whereas IR700 fluorescence from nonspecific tumor vessel volume and/or blood pools is distributed similar to in normal tissues (Fig. 3a, b); based on these findings, we irradiated NIR light for IR700-localizing tumors on Day 1 after Tra-IR700 injection.

Finally, as mentioned above, our previous studies demonstrated that one-time PIT treatment (i.e. mAb-IR700 injection followed by NIR light irradiation) significantly induced target-specific tumor cell death; however, tumor recurrences were eventually seen [12, 14]. Consistent with these previous studies, in the present study, one-time PIT treatment for NCI-N87 tumors with Tra-IR700 was found to significantly suppress tumor growth compared to in the non-treatment control. Furthermore, when 5-FU treatment was added to the PIT treatment, significant tumor growth inhibition compared to PIT treatment alone was observed even in the long-term, indicating a synergistic treatment effect of photoimmunochemotherapy. This combined treatment effect can be explained by the different mechanisms of tumor cell death induced by PIT and 5-FU. The results of our previous studies showed that PIT mainly induces rapid necrotic cell death in response to NIR light irradiation [12–14], whereas 5-FU, as well as trastuzumab, induces apoptotic cell death [23, 24]. Accordingly, whereas only subtle apoptotic changes were observed in the tumors treated with 5-FU alone, pathological analysis showed massive necrotic damage and scant tumor cells with nuclear swelling in the tumors treated with PIT and 5-FU (Fig. 4c).

There are some limitations associated with this method. In clinical cases, the efficacy of Tra-IR700 mediated PIT is limited to gastric cancers because overexpression of HER2 only account for approximately 20 % of all gastric cancer patients [9, 10], however, this study suggests that the Tra-IR700 mediated PIT and PIT in combination with 5-FU could become effective therapies for HER2-positive cases. Furthermore, because of the inhomogeneity of antibody microdistribution in the tumors, NIR light might not be effective for eradicating whole tumor cells, even if those cells express the target molecule abundantly. In addition, the effects of mAb monotherapy on epithelial cancers, such as gastric cancer, are somewhat limited, owing to molecular heterogeneity, and small molecule chemotherapeutic compounds should be considered to improve the antitumor effects in these cases. Furthermore, IR700 is relatively hydrophilic compared to the traditional photosensitizers used for photodynamic therapy, and PIT is considered more effective and less harmful than conventional therapies, mainly owing to the fact that the mAb-IR700 pharmacokinetics allow better specific tumor uptake of IR700 and longer wavelength excitation, thereby allowing better tissue penetration [25–28]. Nevertheless, the limited depth of penetration of NIR light is the primary limitation of this method for clinical gastric cancers. Endoscopic NIR light irradiation could achieve sufficient tissue penetration and improve local tumor control for localized lesions of gastric neoplastic mucosa. In addition, laparoscopic NIR irradiation could improve clinical applicability for disseminated peritoneal gastric cancer and it is further supported by a previous study that demonstrated Tra-IR700 mediated PIT induced significant HER2-targeted tumor killing effect in a mouse model of disseminated peritoneal gastric cancer [29]. However, although the light power is reduced with increasing depth from the light source as a result of light scattering and absorption by the tissues, utilizing a chemotherapeutic agent, such as 5-FU in this case, may still allow tumor cell killing in the deeper tissues where the light is not strong enough to activate the photosensitizer.

## Conclusions

We here found that treatment with PIT in combination with 5-FU resulted in an enhanced antitumor effect through different mechanisms of tumor cell death, as compared to PIT monotherapy, for HER2-expressing human gastric cancer in vitro and in vivo. Immediate target-specific necrotic cell death was induced with one-time administration of Tra-IR700 followed by NIR light irradiation, as determined by HER2-specific IR700 fluorescence imaging, and subsequent 5-FU administration was found to enhance this antitumor effect, thereby effectively inhibiting tumor recurrence. Taken together, our results suggest that photoimmunochemotherapy represents a promising method for treating human gastric cancer, and further studies should be conducted to evaluate whether these results translate into the clinical setting.

## Additional file

Additional file 1:
**Figure S1.** LIVE/DEAD assay. **Figure S2.** Caspase-3 activity assay. (DOCX 397 kb)
