# Supplementary material for: Molecular targeted photoimmunotherapy for HER2-positive human gastric cancer in combination with chemotherapy results in improved treatment outcomes through different cytotoxic mechanisms
Source: BMC Cancer. 2016 Jan 25;16:37. doi: 10.1186/s12885-016-2072-0 (PMC4727331; doi:10.1186/s12885-016-2072-0)
Supplement: Additional file 1: — Figure S1. LIVE/DEAD assay. Figure S2. Caspase-3 activity assay. (DOCX 397 kb) [file 12885_2016_2072_MOESM1_ESM.docx]

**Supplemental Figures and Legends**

**Molecular Targeted Photoimmunotherapy for HER2-positive Human Gastric Cancer in Combination with Chemotherapy Results in Improved Treatment Outcomes Through Different Cytotoxic Mechanisms**

Kimihiro Ito^1^, Makoto Mitsunaga*, Seiji Arihiro, Masayuki Saruta, Mika Matsuoka, Hisataka Kobayashi^2^ and Hisao Tajiri^1^

^1^ Division of Gastroenterology and Hepatology, Department of Internal Medicine, The Jikei University School of Medicine, Minato, Tokyo 105-8461, Japan

^2^ Molecular Imaging Program, Center for Cancer Research, National Cancer Institute, NIH, Building 10, RoomB3B69, MSC1088, Bethesda, MD 20892-1088, USA


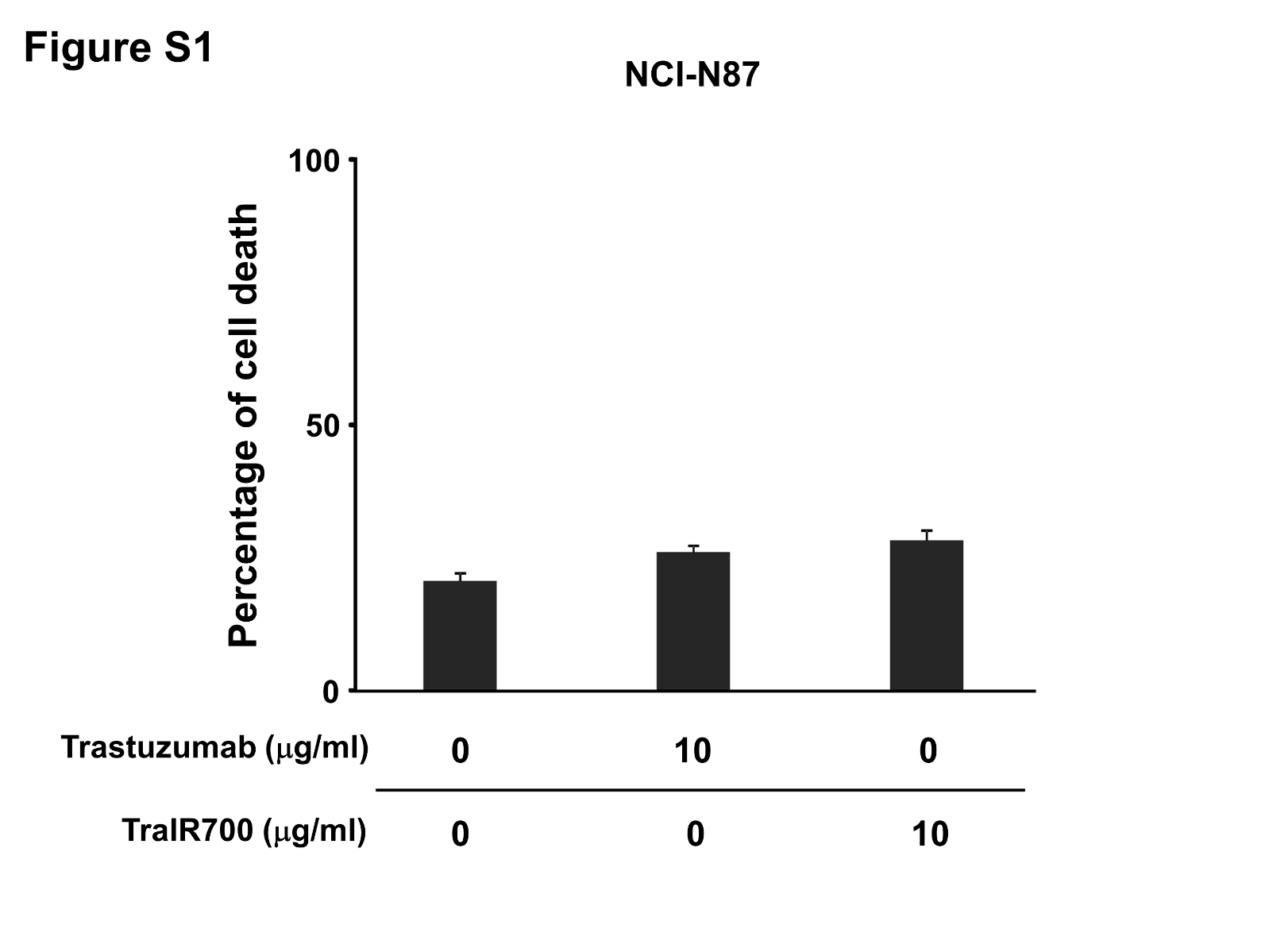


**Figure S1. LIVE/DEAD assay.** There was no significant differences in the percentage of cell death between unconjugated trastuzumab and Tra-IR700 treatment in HER2-expressing NCI-N87 cells. Data are presented as means ± SEM (*n* = 3, Student’s *t* test)

**
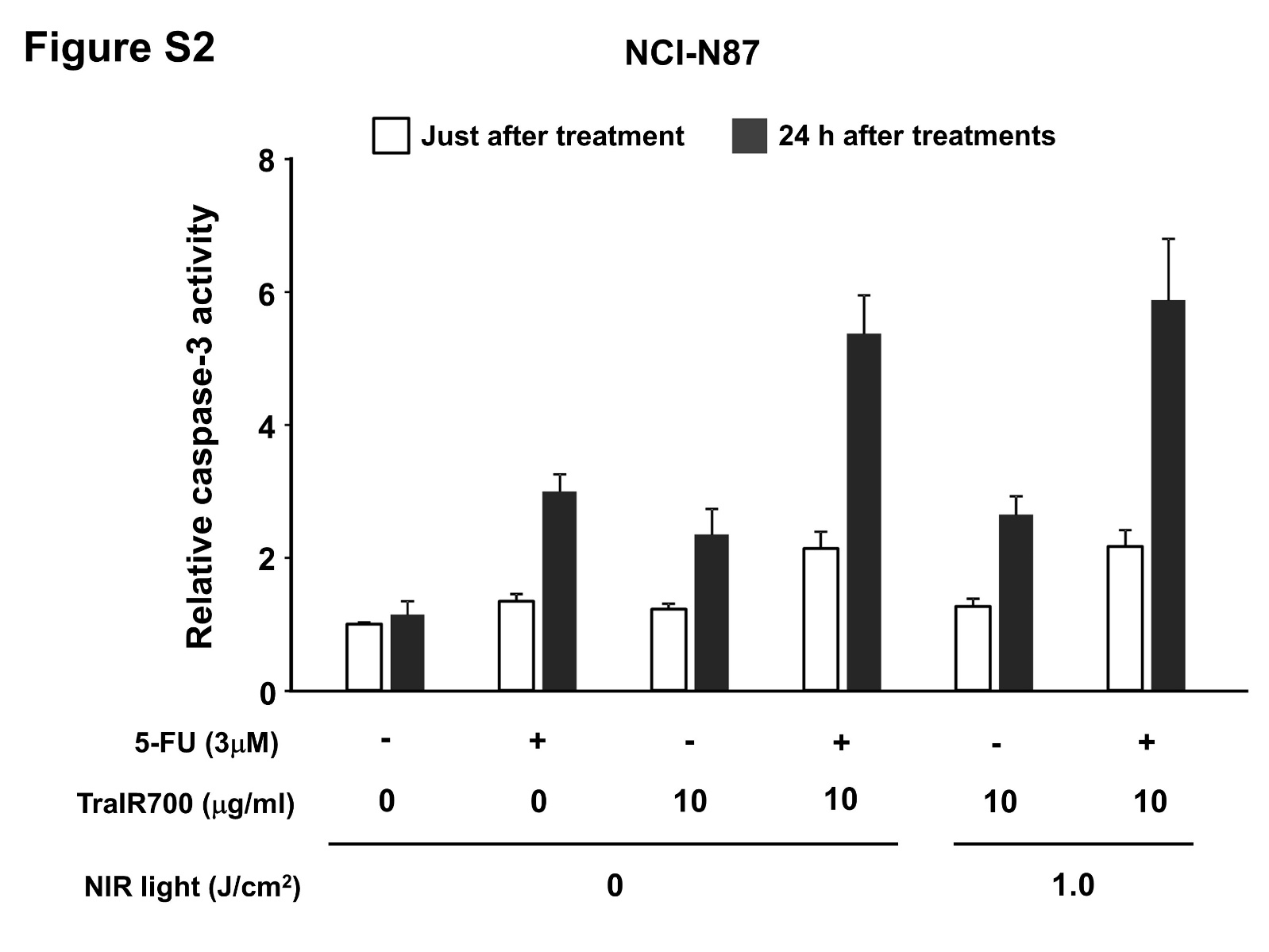
**

**Figure S2. Caspase-3 activity assay.** Caspase-3 activity assay were performed at different time points after treatment. NIR light irradiation after incubation with Tra-IR700 or Tra-IR700 in combination with 5-FU did not result in any additional increases in caspase-3 activity compared to Tra-IR700 treatment (without NIR light) or Tra-IR700 in combination with 5-FU treatment (without NIR light) respectively, just after NIR light irradiation and 24 h after the irradiation. Data are presented as means ± SEM (*n* = 3, Student’s *t* test)
